# Dependence of image quality of late gadolinium enhancement MRI of left atrium on number of patients imaged: results of multi-center trial DECAAF

**DOI:** 10.1186/1532-429X-16-S1-P146

**Published:** 2014-01-16

**Authors:** Sathya Vijayakumar, Eugene G Kholmovski, Mark M Haslam, Nathan Burgon, Nassir F Marrouche

**Affiliations:** 1 Surgical Services Division, Intermountain Healthcare, Salt Lake City, Utah, USA; 2 CARMA Center, University of Utah, Salt Lake City, Utah, USA; 3 UCAIR, Dept. of Radiology, University of Utah, Salt Lake City, Utah, USA

## Background

High-resolution late gadolinium enhancement (LGE) MRI is used to assess fibrosis of the left atrium (LA) and visualize post-ablation scar in patients with atrial fibrillation (AF). Only few centers with advanced expertise in cardiac MR (CMR) have shown successful and good quality LGE-MRI of the LA. In this work, we assess the dependence of image quality of LGE images on the number of patients imaged in the centers participating in the multi-center trial DECAAF (Delayed Enhancement - MRI determinant of successful Catheter Ablation of Atrial Fibrillation). Also, main causes of poor image quality were determined.

## Methods

Fifteen centers with different degrees of CMR expertise and typical MRI hardware participated in DECAAF. Customized sequences for LGE of LA were installed on 17 Siemens scanners in participating centers. Nine centers used 1.5T scanners, five used 3T scanners and one used both 1.5 and 3T scanners. Three hundred and twenty nine AF patients underwent LGE-MRI prior to ablation to estimate the extent of LA fibrosis. One center (8 patients) was excluded from analysis because of two-years of prior experience in LGE-MRI of LA. Two independent readers assessed the image quality as 1- poor, 2 - fair or 3 - good. Poor quality images (57 patients, 17.3%) were analyzed to identify the causes for scan failure.

## Results

**Figure 1:**
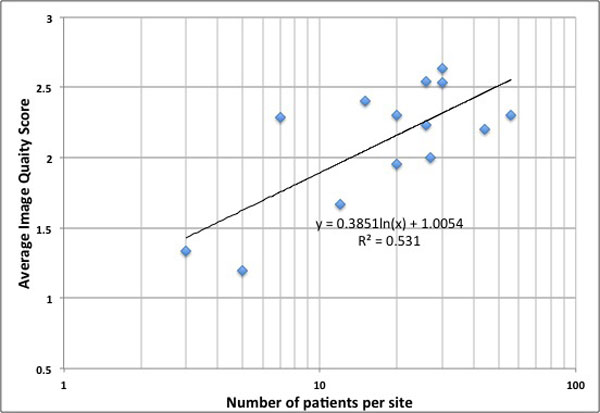
**Dependence of image quality of LGE-MRI on number of patients imaged**.

Figure 1 shows the dependence of image quality on the number of patients imaged in each center. It was found that the average image quality score for centers that had imaged more than 20 patients (2.35 ± 0.23) was statistically significantly higher than the centers that had imaged less than or equal to 20 patients (1.88 ± 0.49) with p < 0.05. Analysis of poor quality images indicated that in 63% of these scans MRI technologist error like wrong inversion recovery time, incorrect navigator prescription, wrong phase encode direction, incomplete coverage of the LA, was the main reason for poor image quality. In 30% cases, poor quality was patient related like significant arrhythmia, very irregular respiration, fast heart rate (> 120 bpm) and patient motion. Hardware limitations were responsible for 7% poor cases.

## Conclusions

The analysis of data from multi-center study DECAAF clearly shows a learning curve associated with LGE MRI of LA - imaging more patients improves image quality. Better training of MRI technologists may also further improve image quality.

## Funding

This study was supported by the CARMA Center at the University of Utah.

